# Redox modulation of muscle mass and function

**DOI:** 10.1016/j.redox.2020.101531

**Published:** 2020-04-18

**Authors:** M.C. Gomez-Cabrera, C. Arc-Chagnaud, A. Salvador-Pascual, T. Brioche, A. Chopard, G. Olaso-Gonzalez, J. Viña

**Affiliations:** a Freshage Research Group, Department of Physiology. Faculty of Medicine, University of Valencia and CIBERFES, Fundación Investigación Hospital Clínico Universitario/INCLIVA, Valencia, Spain; b INRA, UMR866 Dynamique Musculaire et Métabolisme, Université de Montpellier, F-34060, Montpellier, France; c Department of Integrative Biology, University of California, Berkeley, CA, 94705, USA

## Abstract

Muscle mass and strength are very important for exercise performance. Training-induced musculoskeletal injuries usually require periods of complete immobilization to prevent any muscle contraction of the affected muscle groups. Disuse muscle wasting will likely affect every sport practitioner in his or her lifetime. Even short periods of disuse results in significant declines in muscle size, fiber cross sectional area, and strength. To understand the molecular signaling pathways involved in disuse muscle atrophy is of the utmost importance to develop more effective countermeasures in sport science research.

We have divided our review in four different sections. In the first one we discuss the molecular mechanisms involved in muscle atrophy including the main protein synthesis and protein breakdown signaling pathways. In the second section of the review we deal with the main cellular, animal, and human atrophy models. The sources of reactive oxygen species in disuse muscle atrophy and the mechanism through which they regulate protein synthesis and proteolysis are reviewed in the third section of this review. The last section is devoted to the potential interventions to prevent muscle disuse atrophy with especial consideration to studies on which the levels of endogenous antioxidants enzymes or dietary antioxidants have been tested.

## 1 Introduction

Plasticity describes the ability of muscle to adapt to variations in activity and in working demand. The expression became popular since its introduction by the German researcher, Dirk Pette, in 1979. The adaptive event involves the whole muscle fiber structure from myofibrils to mitochondria, membranes, extracellular matrix, as well as capillaries surrounding the muscle fiber [1].

The absence or a reduction in mechanical load results in skeletal muscle atrophy. Atrophy has been defined as a decrease in the size of a tissue or organ due to cellular shrinkage [2]. The decrease in cell size is caused by the loss of organelles, proteins, and cytoplasm. A “normal” mechanical loading pattern is essential to maintain baseline muscle mass [3] and skeletal muscle adapts to a prolonged physical inactivity by decreasing muscle fiber size. On the contrary, mechanically overloaded muscles through synergists ablation, tenotomy or resistance exercise results in skeletal muscle hypertrophy [3].

Mechanosensors allow muscle fibers to sense mechanical forces and trigger the signals involved in the regulation of skeletal muscle mass [4]. There are several identified mechanosensors in the skeletal muscle. Most prominent among them are costameres (dystrophin-glycoprotein and the vinculin-talin-integrin complexes), titin, filamin-C, and Bag3 [3]. It is hypothesized that the activation of these mechanosensitive proteins regulate protein turnover through interaction with the main proteolytic pathways: the proteasome and the autophagic-lysosomal systems, and even with the mammalian target of rapamycin complex 1 (mTORC1), the main nutrient energy sensor controlling protein synthesis (See section 2) [3].

Atrophy is a debilitating response, not only to inactivity [5], but also to many systemic diseases such as hyperuremia [6], chronic obstructive pulmonary disease [7], diabetes [8], sepsis [9], obesity [10], aids [11], cancer [12], and heart failure [13]. Loss of muscle mass, including the loss of muscle fibers, is a common feature in these pathologies in which an activation of the immune and inflammatory response has been widely described [14]. The loss of muscle mass is accompanied by a loss of muscle function and quality in many of the previously mentioned disorders. Muscle quality, is defined as the force generated by each volumetric unit of muscle tissue [15].

Aging is the greatest risk factor for the major chronic musculoskeletal disorders, osteoarthritis, osteoporosis, and sarcopenia [16]. Sarcopenia is a geriatric syndrome, recently considered as a disease, which is associated with low muscle strength, low muscle quantity, and low physical performance [17].

Muscle mass depends on protein turnover and cell turnover that are under the control of different pathways [18]. Cellular turnover plays a major role during muscle development in embryo and in postnatal muscle growth; while protein turnover is dominant over cellular turnover during acute phases of muscle wasting when sarcomeric proteins are rapidly lost i.e. fasting, disuse, and denervation [14]. Satellite cells-mediated myonuclear accretion have a major role during maturational skeletal muscle growth that persist into late adolescence [19] and during acute injury-induced skeletal muscle regeneration [20]. However, the contribution of cellular turnover and of satellite cells to the homeostasis of adult fibers is minor, and its role in the regulation of muscle mass has been questioned by several experimental evidences [14,19].

Loss- and gain-of function studies that include the development of conditional satellite cell specific Knock-Out (KO) mice [[21], [22], [23], [24]], have shown that satellite cells are not required for the homeostatic maintenance of muscle fiber size in adult or old mice under non stressed conditions [19]. Short term deletion of satellite cells in adult mice does not result in muscle fiber atrophy and sarcopenia is generally not exacerbated. Moreover, satellite cells depletion does not cause or worsen muscle fiber atrophy during unloading neither hampers regrowth during reloading [19,25]. On the contrary, genetic modifications that interfere with embryonic and postnatal growth result in smaller muscles in adults. But this reduction in muscle size is caused by failure/inhibition of growth and not by a real atrophy process [14].

## 2 Molecular mechanisms involved in muscle atrophy

Disuse muscle atrophy is due to both a decrease in protein synthesis and an increase in protein breakdown [26,27]. Muscle protein synthesis declines within 6 h following muscle inactivity and it is accompanied with a large increase in muscle protein breakdown [28].

As mentioned in the introduction, the turnover of contractile proteins depends on mechanical stress, nutrients availability, hormones, and growth factors. Thus, aging, physical inactivity, and systemic diseases are well-known modulators of this balance [14,29].

Muscle atrophy is an active process controlled by transcriptional programs [14]. In this section we will review the main molecular mechanism and specific signaling pathways involved in the process.

### 2.1 IGF1-Akt-FoxO signaling pathway

The main pathway involved in protein synthesis and in the regulation of skeletal muscle mass is the highly conserved signaling pathway initiated by IGF1-PI3K-Akt [29]. The binding of insulin, growth factors or amino acids to IGF1 receptor results in the activation of PI3K. It consequently increases Akt activity which stimulates protein synthesis via mTOR [30]. Final targets of mTOR as 4E-BP1, S6K1 or eukaryotic initiation factors (eIF3F, eIF2α) allow ribosomal biogenesis and protein translation [31]. Besides its ability to stimulate protein synthesis, Akt can depress protein degradation through the inhibition of class O type of forkhead transcription factors (FoxO) family [32]. Indeed, Akt-mediated phosphorylation of FoxO1, FoxO3a, and FoxO4 inhibits FoxO-dependent transcription responsible of various cellular process such as autophagy and protein breakdown [32]. Disuse muscle atrophy is characterized by lower rates of protein synthesis due to a downregulation of some mTOR actors, such as Akt, S6K1 or eIF2-α [33,34]. Moreover, four distinct pathways involved in protein degradation are usually up-regulated in disuse muscle atrophy: the calpain system, apoptosis, the autophagic-lysosomal system, and the ubiquitine proteasome pathway. They will be reviewed in the following sections.

### 2.2 Calpain system

Calpains are calcium-dependent, non-lysosomal cysteine proteases, located in the Z disks in skeletal muscle [35]. Desmin, tropomyosin, troponin T, troponin I, and titin are among the myofibrillar proteins digested by the two types of calpains identified in the muscle (type I and type II) [36]. Calpain-mediated proteolysis of myofibrillar components is activated in muscle wasting conditions contributing to the loss of skeletal muscle mass [35].

### 2.3 Mitochondria as a source of catabolic signals

Mitochondria are critical in regulating myofiber metabolism and play a key role in apoptosis [37]. Apoptosis, both intrinsic (involving mitochondria) and extrinsic (involving for instance TNF-α) increases dramatically during the early phase of atrophy [38,39]. Mitochondria releases pro-apoptotic factors into the cytosol, such as the B-cell lymphoma (Bcl)-2 family proteins or cytochrome c [37]. These mitochondrial proteins activate numerous caspases [40]. Caspases are thought to be the main proteins involved in both the triggering (caspase-8, -9, -12) and execution of apoptosis (caspase-3, -6, -7) [41]. Their enzymatic action let cleavage of target proteins of the nuclear envelope and DNA [41]. More specifically, mitochondrial protein-induced caspase-3 activation has been identified as a critical event in apoptosis [42] an in myofiber's atrophy, through the degradation of actomyosin complexes [43]. Interestingly, mitochondria also regulate apoptosis through a caspases-independent mechanism, which relies on the release of mitochondrial proteins into the nucleus, especially apoptosis-inducing factor (AIF) and endonuclease G (EndoG) [41].

### 2.4 Autophagy-mediated protein breakdown

The autophagic-lysosomal system is a catabolic process emerging as a major regulator of muscle mass. It recycles damaged organelles and generates metabolic substrates necessary to the maintenance of basal cellular activity [44]. Autophagy is the only pathway able to massively degrade macromolecules and organelles [45]. It relies on the action of two vesicles, the autophagosome, which captures the substrates, and the lysosome, that fuse with the autophagosome and degrades it with its constituents [46]. The complex Ulk1-Atg13-FIP200 plays a key role in the initiation of autophagy. Ulk1 can be phosphorylated by AMPK on ser^777^ and ser^757^, leading to its activation, whereas its phosphorylation by mTORC1 leads to its inhibition [47]. AMPK acts as the sensor of energy balance and is a key factor in the regulation of myofiber size [14]. Indeed treating muscle cell cultures with an activator of AMPK (AICAR) causes an increase in proteolysis and in the expression of MuRF-1 and MAFbx via the FoxO family [48]. Autophagosome formation involves the action of various Atgs (autophagy related genes) and especially LC3, essential for the elongation and formation of a mature autophagosome [49]. Autophagy is essential in muscle homeostasis maintenance. Its deficit triggers damaged proteins accumulation leading to muscle atrophy and in some cases, myopathies [44]. However, the overactivity of the autophagic system can also lead to amyotrophy and muscular pathologies [50]. To summarize, both excessive and defective autophagy are highly associated with skeletal muscle loss.

### 2.5 Ubiquitin-proteasome system

The ubiquitin-proteasome system is a protein degradation pathway that plays a key role is skeletal muscle atrophy [27]. This ATP-dependent system involves the binding of ubiquitin on proteins’ lysine residues. These poly-ubiquitinated substrates are directed to the proteasome, which will be in charge of their degradation into peptides [51]. This pathway includes three critical atrogenic muscle-specific E3 ubiquitin ligases: muscle RING Finger 1 (MuRF-1), muscle atrophy F-box (MAFbx), and Casitas B-lineage lymphoma b (Cbl-b) [52,53]. They regulate the degradation of skeletal muscle proteins such as calcineurin, myoD, troponin-I, titin, myosin heavy and light chains, and the IGF-1 signaling intermediate insulin receptor substrate-1 (IRS-1) [54]. The ubiquitin-proteasome system that is constitutively operative in normal skeletal muscle, is responsible for the turnover of most soluble and myofibrillar muscle proteins due to changes in muscle contraction [55]. The activity of this pathway is markedly increased in atrophying muscle due to transcriptional activation of ubiquitin, of several proteasomal subunit genes, and of the ubiquitin ligases [26]. Importantly, the rate of muscle atrophy is markedly reduced by targeted inactivation of these gene products [53].

### 2.6 Inflammatory cytokines and NF-κB signaling

Inflammation and oxidative stress are two common mechanisms in disuse muscle atrophy [56,57]. Pro-inflammatory factors favor protein breakdown through the activation of MuRF-1 and MAFbx, via NF-κB, Fox O, and p38MAPK signaling pathways [58].

NF-κΒ is considered as one of the main inflammatory pathways influenced by plenty of cytokines, chemokines, and adhesion molecules [59]. More precisely, IL-1 and TNFα pro-inflammatory cytokines stimulate NF-κB by the intermediate of IKKβ/α activation [60]. Moreover, chronic increase of circulating IL-6 levels induce the activation of JAK/STAT catabolic pathway and also downregulate S6 kinase phosphorylation, both contributing to muscular atrophy [61]. Since the beginning of this century, research has provided evidence that inflammation has a synergistic link with oxidative stress, controlling skeletal muscle mass and function particularly in a great number of chronic diseases, as well as in cancer [62]. Literature abounds of studies demonstrating an association between oxidative stress and muscular atrophy [[63], [64], [65], [66]]. The molecular mechanisms involved will be described in Section 3.

### 2.7 Myostatin and muscle atrophy

An important negative regulator of muscle growth is myostatin, also known as Growth Differentiation Factor-8 (GDF-8). Myostatin is expressed in developing skeletal muscles in the embryogenesis to regulate the number of muscle cells. During adulthood its production by skeletal muscle limits fiber hypertrophy [67].

Myostatin KO mice exhibit an hypertrophic phenotype due to an increase in muscle fiber size and number [68]. Despite of their increased muscle mass, these animals have an alteration in their myofibers’ contractile properties which results in a low force and power generation [69] and a higher muscle fatigue [70]. Consistent with these results, it has been shown that myostatin reduces the Akt/TORC1/p70S6K signaling, inhibiting myoblast differentiation and myotube size [71]. Although the molecular mechanism of myostatin-mediated cellular effects are not totally elucidated, the involvement of the transcription factors Smad2 and Smad3 [71] and of the FoxO family of proteins has been suggested [14]. As a summary, myostatin inhibition induces muscle hypertrophy while local administration of myostatin triggers muscle atrophy and decreases skeletal muscle force generation [69, 71].

## 3 Skeletal muscle atrophy models

### 3.1 Cellular models

**Fig. 1:**
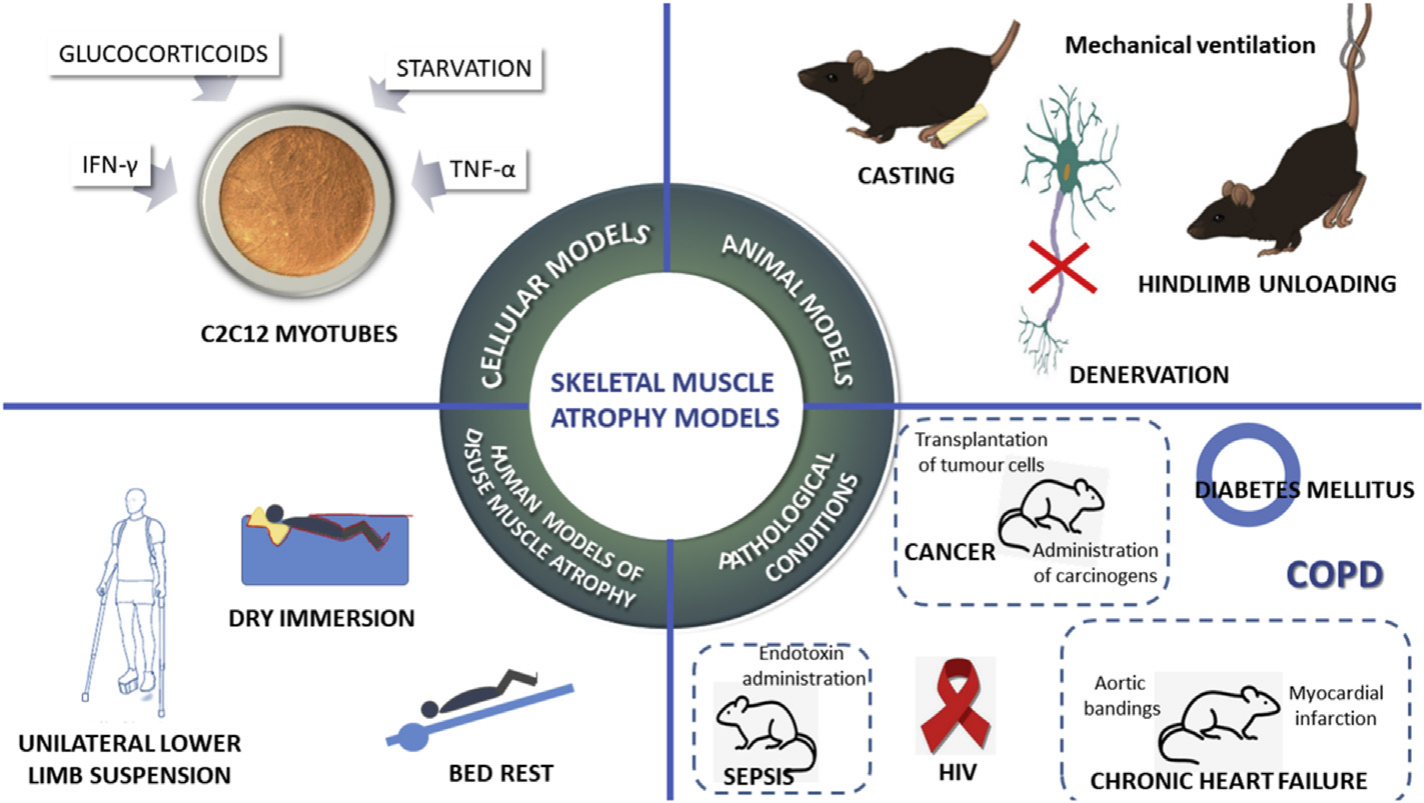
Skeletal muscle atrophy models. C2C12 myotubes are the main cellular model used to study atrophy *in vitro*. Among the main disuse muscle atrophy animal models, we can highlight: denervation, casting, hindlimb unloading and mechanical ventilation. Four models are validated by the scientific community to study disuse muscle atrophy in humans. Atrophy is a debilitating response, not only to inactivity, but also to many systemic diseases such as sepsis, HIV, cancer, chronic obstructive pulmonary disease (COPD), diabetes mellitus, and heart failure.

To investigate the molecular pathways involved in muscle atrophy, several cell culture models have been developed (See Fig. 1). Starvation of cultured cells is a very common experimental method to trigger atrophy in myotubes [29]. In this model, cells are deprived of nutrients by replacing their culture media by Phosphate-Buffered Saline leading to a severe atrophy [18]. In such cultured myotubes, Akt is inhibited and FoxO and MAFbx gene expression, severely activated [32].

Pro-inflammatory cytokines (such as TNF-α or IFN-γ) [72] and glucocorticoids (such as dexamethasone and corticosterone) [73], are also able to induce *in vitro* atrophy through an increase in proteolytic and apoptotic signals.

### 3.2 Animal models of skeletal muscle atrophy

Rodents, predominantly rats and mice, are widely used models to study disuse muscle atrophy. Mechanical ventilation (MV), denervation, casting, and hindlimb unloading (HU) are the four main models to induce muscle wasting [74] (See Fig. 1).

One of the most widely studied models of disuse in animals, but also in humans [75], is mechanical ventilation. MV is a widely used model for its importance in clinical practice [66]. It induces, in a matter of hours, a significant diaphragm atrophy and loss of force that is accompanied by an increase in oxidative stress [76]. Briefly in the MV model the animals are tracheostomized and the diaphragm is completely inactive because a mechanical ventilator delivers all breaths [77].

*Denervation* consists on the removal of nerve supply leading to the loss of muscle contraction's capacity due to the lack of nervous stimulation. It triggers rapid deleterious effects on muscle tissue associated with an activation of multiple proteolytic systems [78]. Reductions in the cross-sectional area (CSA) of the fibers accompanied by a loss of maximal strength have been reported in denervated muscles [79,80].

Another model used to study muscle atrophy is the *casting protocol*. Usually, one hindlimb is casted to induce atrophy and the contralateral acts as control [81].

In the mid-seventies, the National Aeronautics and Space Administration started using the HU model. Its use in numerous projects confirmed its relevance in the study of weightlessness in muscle deconditioning [82,83]. The HU model consists of a slight head-down inclination of the animal (about 30%) using tail or pelvic suspension. Thus, hindlimb do not reach the ground but animals are free to move, eat, and groom with their forelimbs [82]. Besides inducing a cephalad fluid shift typical of microgravity conditions, this model results in the loss of muscle mass [84,85]. Soleus muscles are especially affected by HU-induced atrophy, with a decrease in muscle force, together with a slow-to fast transition characterized by an overexpression of the fast Myosin Heavy Chain (MHC) isoforms [86]. A novel hindlimb partial gravity quadrupedal unloading model in rats has been recently suggested for investigating the physiological alterations occurring in partial gravity environments [87]. More research is needed to stablish whether this method improves the existing ones.

### 3.3 Skeletal muscle atrophy in humans

Although cellular and animal models are useful to understand muscle wasting mechanisms, the development of experimental models in humans is of the utmost importance. Four models are validated by the scientific community to study disuse muscle atrophy in humans: unilateral lower limb suspension (ULLS), head-down bed rest (HDBR), dry immersion (DI), and microgravity experiments (See Fig. 1).

The first study using *ULLS* was published 30 years ago [88]. Briefly, one of the legs is maintained in suspension thanks to an elevated sole of a shoe, which eliminates ground contact on the adjacent foot. It unloads the lower limb but allows ankle, knee, and hip joint mobility. This model is closely linked to clinical aspects of immobilization following joint or skeletal injuries. The main features of ULLS are: loss of muscle mass, loss of muscle strength [88,89] and increase in intermuscular adipose tissue accumulation [90]. This is accompanied by an increase in the proteolysis rates of unloaded muscles [91] and a selective decrease in the CSA in both type I and IIa myofibers [92].

In the *HDBR* model, subjects are inclined by −6° in a supine position to induce an upward fluid shift characteristic of microgravity conditions. It is a reliable simulation model for most of the physiological effects of spaceflight, and allows the study of countermeasure interventions [93]. HDBR is characterized by i) muscle weakness and low muscle power; ii) loss of muscle mass especially in knee and ankle extensor muscles [94,95]; iii) a myotypologic shift from slow-to-fast MHC; iv) a presence of hybrid fibers [96,97]; v) a reduction of myofibrillar protein content [98].

In the 70's, with the emerging of space programs, soviet researchers introduced *DI* as a new weightlessness simulation method [99]. This experimental model consists in the immersion of a subject in a thermoneutral water covered with an elastic waterproof fabric. It faithfully mimic spaceflight through centralization of body fluids, unloading, hypokinesia, and the lack of a supporting structure under the body [100]. DI reduces the mechanical stress in skeletal muscle leading to a faster reduction in muscle tone and tension when compared to bed rest [100]. Loss of muscle mass and force are evident after only few days of DI in vastus lateralis and soleus muscle [101]. An increase of intermuscular adipose tissue and adipogenic markers have also been found in short-term protocols of only 3 days duration [102].

Finally, real *microgravity experiments* largely contribute to data collection and knowledge progress regarding muscle deconditioning. On-board experiments with rodents in satellites, as well as experiments in the orbital International Space Station, provide the opportunity to test countermeasures, most of them based on exercise or nutritional interventions, to prevent disuse muscle atrophy [103].

## 4 Oxidative stress as a common mechanism in different atrophy models

As mentioned in a previous section, inflammation and oxidative stress are two common and interrelated mechanisms in disuse muscle atrophy [56]. Several evidences indicate that the activation of redox-sensitive transcription factors, such as NF-κB, may modulate the gene expression of key players involved in the inflammatory response, IL-1β, IL-6, (COX)-2, adhesion molecules, inducible nitric oxide (NO) synthase (iNOS), and TNF-α [104]. The relationship between TNF-α and the generation of reactive oxygen species (ROS) in skeletal muscle has been well described (Reid & Moylan, 2011). TNF-α activates the TNF-1 receptor in the sarcolemma, initiating a signaling cascade that leads to an increase in the mitochondrial production of superoxide ion. Moreover, arachidonic acid, the main precursor of prostaglandins that play a key role in the inflammatory response, increases ROS generation through the activation of NADPH-oxidases (NOXs) and lipoxygenase [105].

In this section, we will mainly focus on the role of oxidative stress in muscle atrophy and the potential countermeasures that, based on in its implication have been tested.

First evidence showing that skeletal muscle contains free radicals was reported in the 50's in Nature [106]. During the 80's researchers identified the first link between muscle contraction and free radical biology when Davies and co-workers showed, for the first time *in vivo*, a 3-fold increase in free radical content of skeletal muscle from rats run until exhaustion [107].

The notion that increased ROS and disturbances in redox signaling play a significant role in the promotion of disuse muscle atrophy was proposed over 30 years ago by a Japanese group using hindlimb unloading in rats [108].

We now know that ROS production occurs at different extents in the diverse atrophy models and muscle types [28]. For instance, the rate of muscle atrophy is extremely fast in diaphragm in MV but slower in soleus and gastrocnemius muscle in HU [109]. Shanely and co-workers first showed, in a rat study, that MV induces an extremely rapid diaphragm atrophy and force loss and that the oxidative stress plays a major role in the phenomenon. More specifically they found that MV was associated with a rise in protein and lipid oxidation in the diaphragm [76]. Shortly afterwards, it was concluded that the primary target of the MV-induced oxidative injury in diaphragmatic proteins were insoluble proteins with molecular masses of 200, 120, 80, and 40 kDa [110]. Dr. Powers research group found that in the diaphragms of MV animals the oxidative stress depends on both an increase in ROS production and a decrease in total antioxidant capacity and in the glutathione levels [111]. Lately, the role of oxidative stress in MV was further refined with the development of antioxidant trials. Although it will be discussed in a later section in the manuscript (See section 4) it has been shown that the administration of a ROS scavenger, N-acetylcysteine (NAC), that provides cysteine for the synthesis of the antioxidant glutathione, prevents against MV-induced diaphragmatic oxidative stress, proteolysis, and contractile dysfunction [112]. It has also been reported that MV induces an increase in the expression in the diaphragm of E3 ubiquitin ligases and autophagy genes and treatment with NAC also prevents it [113]. Finally, a clinical trial in critically ill patients found that the duration of mechanical ventilation in the intensive care unit patients was reduced when they received enteral administration of antioxidants [114].

Paradoxically, both skeletal muscle contraction and disuse or inactivity are associated with an increase in ROS generation leading to very different outcomes in the muscle cell. Contraction-induced ROS are known to be critical in two main muscle adaptations to exercise training in skeletal muscle i.e. mitochondrial biogenesis and the endogenous antioxidant defense [115,116]. However, it has also clearly being demonstrated that the chronic increase in ROS generation in skeletal muscle in disuse models is involved in skeletal muscle atrophy [75]. The mechanisms underlying the opposite effects of ROS on muscle homeostasis in different conditions are still unclear [66].

The next sections will be devoted to summarize the mechanism through which oxidative stress can cause skeletal muscle atrophy.

### 4.1 Sources of ROS in disuse muscle atrophy

**Fig. 2:**
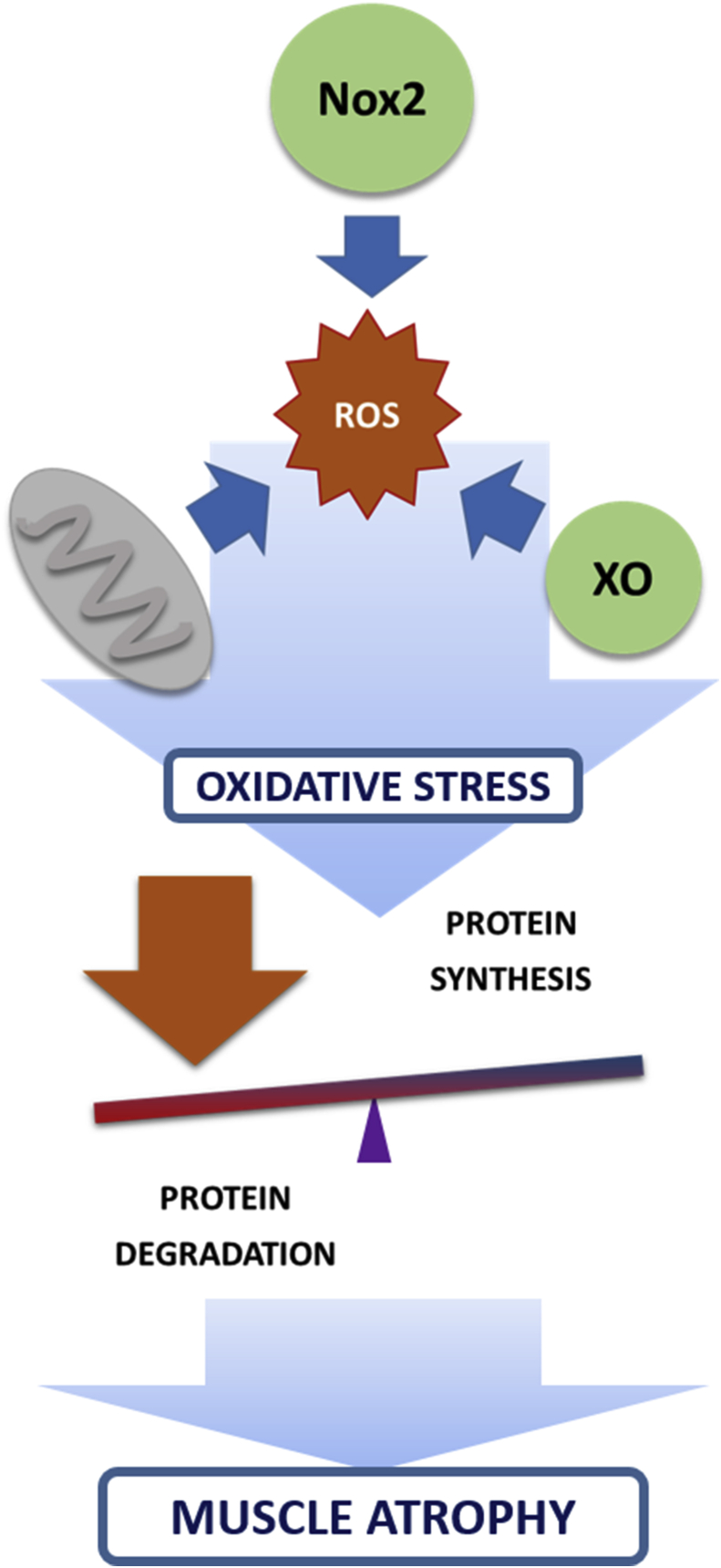
Sources of ROS in disuse muscle atrophy. ROS are generated in different intracellular and extracellular locations in the muscle cell. They can be generated by NADPH oxidases (NOXs) in the sarcolemma and the sarcoplasmic reticulum, by xanthine oxidase (XO) in the skeletal muscle extracellular space, and by mitochondria. Oxidative stress is involved in both a decrease in protein synthesis and an increase in protein breakdown in disuse muscle atrophy.

Although some studies have failed to detect a link between oxidative stress and disuse muscle atrophy [64,109,[117], [118], [119]], many research groups, including ours, have found that prolonged inactivity is associated with oxidative stress in animal and human studies [55,[120], [121], [122], [123], [124]]. ROS are generated in different intracellular and extracellular locations in the muscle cell including the sarcolemma, the sarcoplasmic reticulum, and mitochondria [125] (See Fig. 2).

#### 4.1.1 Mitochondria

Prolonged immobilization is characterized by mitochondrial deterioration and proteolysis [126]. The respiratory capacity of muscle mitochondria is reduced during long periods of muscle inactivity [127] while the ROS generation is increased [123]. Four major hypotheses have been suggested to understand the role of mitochondria as a source of ROS in muscle disuse. First, it has been proposed that the changes in the cardiolipin content and composition in the mitochondria during immobilization can increase ROS generation through the reduction in the activity of cytochrome c oxidase [[128], [129], [130]]. The observation that mitochondrial Ca^2+^ levels are increased during immobilization [131] provides a second line of evidence to support the role of mitochondria as a source of ROS in disuse muscle atrophy. Mitochondrion acts as a sink for the increased cytosolic Ca^2+^ levels in unloaded muscles, due to a leak of this ion caused by the oxidation of ryanodine receptor 1 in the sarcoplasmic reticulum [131]. Once in the mitochondria Ca^2+^ stimulates the proton motive force and increases ROS production [132]. Mitochondrial Ca^2+^ also activates ROS-generating enzymes such as α-ketoglutarate dehydrogenases and glycerolphosphate dehydrogenase [133]. A third line of evidence to support the role of mitochondria in the generation of ROS during prolonged inactivity points to the translocation into the organelle of the signal transducer and activator of transcription 3 (STAT3). When STAT3 is activated, it is able to bind to the mitochondrial complex I subunit thus increasing mitochondrial ROS generation [134]. Finally, transfecting experiments and the use of transgenic animals provide the fourth line of logic to link mitochondrial ROS and muscle atrophy. We and others have shown that preventing the muscle wasting induced downregulation of PGC-1α, the master regulator for mitochondrial biogenesis, protects skeletal muscle from the atrophy induced by unloading [126,135], denervation, fasting or FoxO3 overexpression [136] (See Fig. 2).

#### 4.1.2 NADPH oxidases

Another source of ROS in skeletal muscle are the NADPH oxidases (NOXs) family of enzymes [137]. Only Nox2 and Nox 4 are found in skeletal muscle. They are located in the transverse tubule, mitochondria, sarcolemma and sarcoplasmic reticulum and are able to attach to proteins such as p22^phox^, p67^phox^, p47^phox^, and p40^phox^ [138]. Nox2 and Nox 4 generate mainly O_2_^·-^ and H_2_O_2_ respectively. ROS generated by muscle NOXs enzymes are, at least, partially responsible of the oxidative damage in muscle atrophy [139]. It has been shown that apocynin, the inhibitor of NOX activity, attenuates diaphragm oxidative stress, atrophy, and protease activation during prolonged mechanical ventilation [140]. Moreover, deletion of Nox2 prevents Angiotensin II-Induced skeletal muscle atrophy [141] (See Fig. 2).

#### 4.1.3 Xanthine oxidoreductase

Xanthine oxidoreductase (XOR) is an enzyme involved in purine catabolism [142]. This enzyme catalyzes the oxidation of hypoxanthine to xanthine and can further catalyze the oxidation of xanthine to uric acid [143]. XOR exists in two interconvertible forms. In the oxidase form (XO), molecular oxygen is used as the electron acceptor and hypoxanthine and xanthine are oxidized to uric acid and superoxide radical [144]. First evidence showing an increase in XO activity and oxidative damage in the soleus muscle of immobilized rats was published in 1993 [145] (See Fig. 2). Since then, and by using allopurinol, a well-known inhibitor of XOR, the role of XO as a source of ROS in immobilized rats has been demonstrated [145] in hindlimb unloading [52,146], in MV-induced diaphragmatic contractile dysfunction [147], and in cancer cachexia [148] (See section 4).

### 4.2 Role of oxidative stress in the regulation of protein synthesis

ROS are able to modulate the insulin regulated protein synthesis pathway through the PI3K-Akt-mTOR axis [105]. One of the earliest connections between ROS and insulin was the documentation that millimolar concentrations of H_2_O_2_ could induce the metabolic actions of insulin by activating its signaling thought the modulation of the tyrosine kinase-phosphatase balance [149]. Physiologically relevant concentrations of H_2_O_2_ (<0.1 mM) were found to enhance the cellular response to insulin through the phosphorylation of specific tyrosine in the insulin receptor beta subunit (IRβ), indicating a role of ROS in insulin receptor activation [150].

*In vivo* studies have demonstrated that mitochondrial ROS production, generated during muscle contraction, stimulates the glucose transport into muscle cells during exercise [151]. More specifically and using isolated muscles, it has been shown that an acute exposure of exogenous H_2_O_2_ in the extensor digitorum longus increases glucose transport at doses up to 1.2 mM but higher doses of H_2_O_2_ return glucose transport to basal levels [152]. Consistent with these results Dr. Ristow an co-workers found, in a human study, that an antioxidant cocktail (the combination of vitamins E and C) prevented the exercise training induction of molecular regulators of insulin sensitivity [153]. The authors found that the exercise-induced oxidative stress ameliorated insulin resistance and caused an adaptive response promoting endogenous skeletal muscle antioxidant defense capacity. However, the antioxidant supplementation precluded these health-promoting effects of exercise in humans.

Collectively, these studies suggest that an optimal range of ROS levels may result in the optimal regulation of their physiological functions, including the protein synthesis pathways [149,154].

**Fig. 3:**
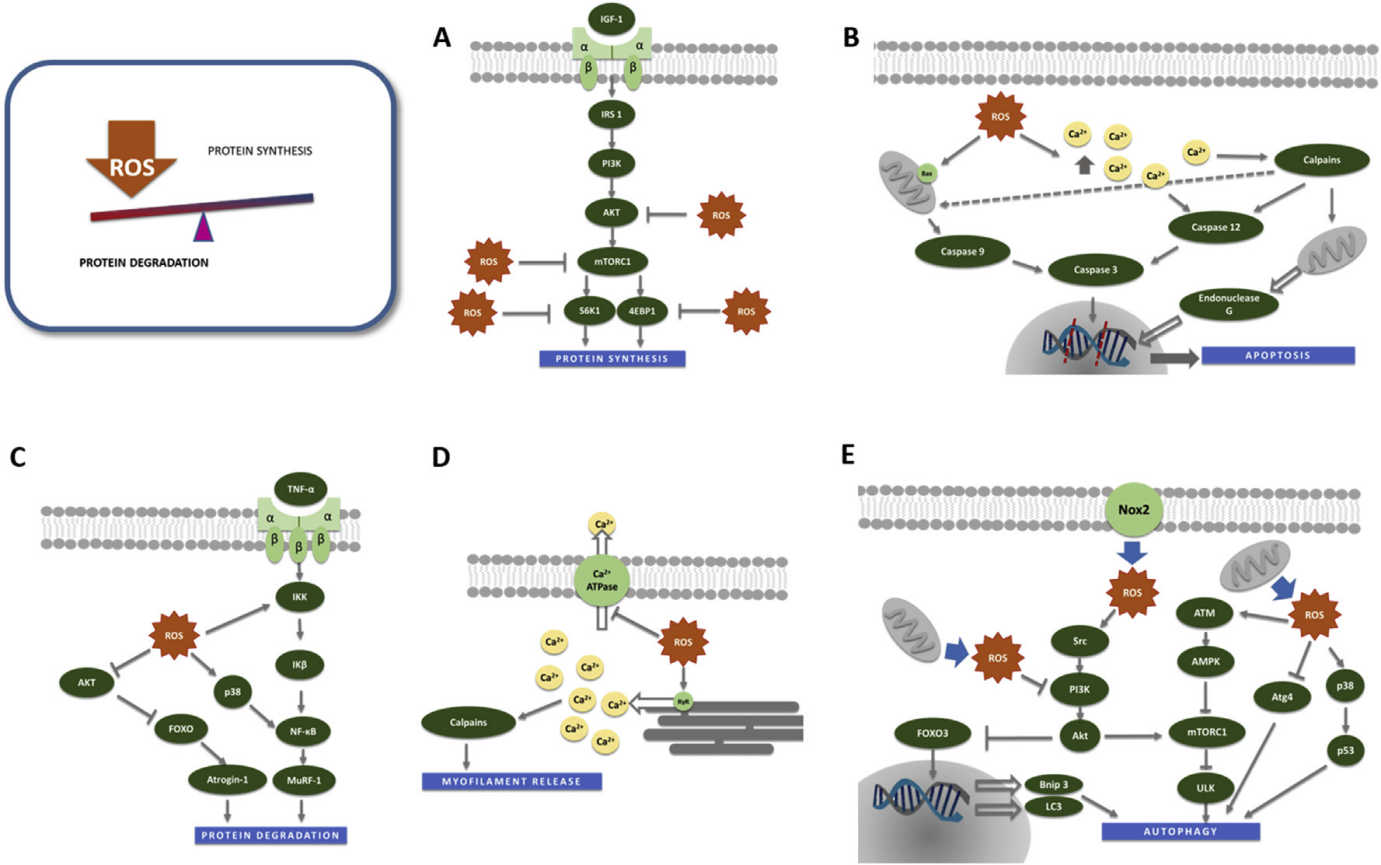
Role of oxidative stress in the regulation of muscle atrophy. **A.** High levels of ROS downregulate the PI3K-Akt-mTORC1 axis by several mechanisms including a decline of Akt phosphorylation, the repression of mTORC1 and the inhibition of 4E-BP1 and S6K1 phosphorylation which are essential for protein synthesis. **B.** ROS activate caspase-3 through the activation of caspase-12 (extrinsic apoptotic pathway) or caspase 9 (intrinsic apoptotic pathway). Calpains also contribute to apoptosis through caspase-12 activation and promoting EndoG pro-apoptotic factor release from mitochondria. **C.** ROS up-regulate the ubiquitin proteasome system by increasing the gene expression of MuRF-1 and MAFbx by FoxO3 dephosphorylation and NF-κB activation. **D.** Oxidative stress promotes the activation of calpains by cytosolic Ca^+2^ increase. **E.** Activation or inhibition of autophagy depends on the sub-cellular ROS localization. Nox2-derived ROS lead to the activation of SCR-PI3K-Akt pathway and inhibit autophagy. However, mitochondrial ROS activate autophagy by i) inducing FoXO3 nuclear translocation, due to the Akt-mTOR pathway inhibition, which leads to autophagy related genes expression; ii) repressing mTORC1 by ATM activation which increases ULK activity; iii) inducing p38/p53; iv) inactivating ATG4 which prevents the premature cleavage of LC3.

It has been suggested that ROS could interfere with protein synthesis through the following mechanism: i) inhibition of the mRNA translation initiation process [155]; ii) inhibition of mTOR by 4E-BP1 and S6K1 phosphorylation [156]; iii) interference with the phosphorylation of Akt [154]; iv) repression of mTORC1 through the accumulation of oxidative DNA damage [157] (See Fig. 3A).

### 4.3 Role of ROS in the regulation of proteolysis

Oxidative stress has also a key role in proteolysis by regulating protein degradation at different levels. We will review the main proteolytic systems modulated by ROS in the following sections.

#### 4.3.1 Redox modulation of the ubiquitin proteasome system

The ubiquitin proteasome system is up-regulated by ROS through the increase in the gene expression of specific ubiquitin-activating enzymes that contribute to muscle protein breakdown such as MuRF-1 and MAFbx [63]. We have found a significant increase in the mRNA levels of MuRF-1 and MAFbx in the soleus muscle of rats and mice after two weeks of HU [55,120]. MAFbx and MuRF-1 expressions are regulated by FoxO [158]. Akt inactivation triggers FoxO3 dephosphorylation and its translocation into the nucleus which is required for MAFbx an MuRF-1 gene expression [29]. High levels of ROS inactivate Akt favoring the expression of the atrogenic E3 ubiquitin ligases [159].

An alternative pathway to increase the proteasome activity by oxidative stress include the allosteric activation of its core proteasome subunit (20S) [63] that can be prevented with the vitamin E analog, Trolox [77,160]. Moreover, oxidized proteins undergo a change in their secondary and tertiary structure which make them more susceptible to enzymatic hydrolysis by the ubiquitin proteasome system.

Finally, NF-κB as a transcription factor modulated by the thiol-disulfide balance [161] and highly inducible in the different disuse models [14], regulates the expression of specific genes of the ubiquitin proteasome system such as MuRF-1 [120,162]. Additionally, NF-κB increases the expression of pro-inflammatory cytokines such IL-6 and TNF-α which contribute to a higher ROS release and consequently a higher activation of the ubiquitin proteasome system, creating a vicious cycle [163,164] (See Fig. 3C).

#### 4.3.2 Redox modulation of autophagy

First direct evidence showing the regulation of autophagy by ROS was reported in 2008 [165]. The authors found that an increase in the skeletal muscle H_2_O_2_ levels, originated by a mutant form of superoxide dismutase 1 (SOD1), resulted in an autophagy mediated weakness and muscle atrophy in mice [165]. *In vitro* experiments performed in C2C12 myotubes treated with H_2_O_2_ also confirm an autophagy activation [166]. Inhibition of autophagy, in Atg7 KO mice, is accompanied by an increase in ROS generation [44,63]. Thus, the exact crosstalk between autophagy and oxidative stress needs further study [167].

Mitochondrial ROS play a major role in the activation of autophagy during atrophy through inhibition of the Akt-mTOR pathway [165,168] (See Fig. 3E). However, it has also been shown that Nox2-derived ROS lead to the activation of SCR-PI3K-Akt pathway leading to an inhibition of autophagy in a dystrophic mice model [167]. This shows the importance of the sub-cellular ROS localization in the activation or inhibition of autophagy [167]. Other pathway described to induce autophagy includes the ATM activation by ROS, which in turn activates AMPK [169] that represses mTORC1 [170]. This last step is required for the induction of autophagy.

Further, mitochondrial ROS have been shown to induce autophagy in a p38 MAPK dependent manner [166]. It seems that p38/p53 not only activates autophagy, but also are involved in a positive feed-back response, because they increase ROS production in cardiomyocytes [171].

Finally, ROS inactivate ATG4 which prevents the premature cleavage of LC3 during autophagosome formation, an essential step in the process of autophagy [172].

It is clear that basal autophagy is needed for the maintenance of the metabolic homeostasis, while oxidative stress and their subsequent activation of autophagy seems to led to weakness and muscle atrophy (See Fig. 3E).

#### 4.3.3 Redox modulation of calpain proteases

Oxidative stress promotes the activation of calpains in several cell types including skeletal muscle [36,173,174] (See Fig. 3D). Treating C2C12 or human myoblasts with H_2_O_2_ activates calpain 1 and calpain 2 [166] due to an increase in cytosolic free-calcium levels [36]. Although the mechanism is not completely elucidated it is considered that ROS could either mediate the formation of reactive aldehydes and inhibit Ca^2+^-ATPasa activity hampering the removal of cytosolic Ca^+2^ [175], or they could oxidize the ryanodine receptor leading to a leak of Ca^+2^ from the sarcoplasmic reticulum to the cytosol [176]. Calpain-dependent proteolysis have also been involved in age-associated loss of skeletal muscle mass [177]. EndoG pro-apoptotic factor can be released from mitochondria by calpain activity [41] (See Fig. 3D). Therefore, since oxidative stress increases the activity of calpains, indirectly, apoptosis may also be increased.

#### 4.3.4 Redox modulation of caspases

Caspase-3 is activated in skeletal muscle under disuse conditions and in C2C12 myotubes treated with H_2_O_2_ [36,178,179]. Caspase-3 is the effector in which converge the extrinsic (mediated by TNF-α or Ang-II) and the intrinsic (mitochondria-dependent) apoptotic pathways. The latter triggers an imbalance between antiapoptotic factors such as Bcl-2 and apoptotic factors such as Bax. In disuse muscle atrophy, Caspase-3 can be activated by Caspase-12 as a result of calcium release from the sarcoplasmic reticulum or by the activation of caspase 9 (through the mitochondrial pathway) [65,180]. In an experimental model of cancer cachexia, it has been shown that by increasing ROS, the expression and mitochondrial translocation of the proapoptotic factor Bax leads to the formation of the mitochondrial transition pore [181] (See Fig. 3B).

## 5 Redox imbalance and disuse muscle atrophy. Potential countermeasures

The role attributed to oxidative stress in regulating the protein synthesis and degradation balance in disuse muscle atrophy open up room for intervention. The main corollary of the ROS hypothesis of skeletal muscle atrophy is that by up-regulating the antioxidants enzymes or by giving antioxidants (vitamin E, vitamin C, carotenoids, α-lipoic acid, polyphenols, N-acetylcysteine, the soybean-derived Bowman-Birk inhibitor, allopurinol or even SS-31) one could prevent disuse muscle atrophy [28]. Regarding the first approach, studies of tissue specific molecular models lacking antioxidant enzymes, have highlighted the potential role that disrupted redox pathways can play in muscle loss and weakness [182]. More specifically, it has been shown that a whole body deletion of SOD1, localized in the cytosol and mitochondrial intermembrane space, plays a role in muscle loss, weakness, and in sarcopenia in mice [183]. Very interestingly, a muscle-specific expression of a mutant SOD1 protein causes muscle atrophy, a decrease in muscle strength and an increase in oxidative stress [165]. These results have led to the consideration of the SOD1 KO mice, as a model of frailty [184]. These mice exhibit skeletal muscle weakness that is accompanied with inflammation, mitochondrial dysfunction, and oxidative stress [184]. On the contrary, a significant number of studies in transgenic mice overexpressing antioxidant enzymes show enhanced health span, reporting the majority of the studies improvements in the cardiovascular and lung function, in neurodegeneration, in cancer, and in diabetes [182]. On this regards, we have shown that the overexpression of the antioxidant enzyme Glucose 6-Phosphate Dehydrogenase (G6PD) in mice, protects from the age-associated oxidative damage in different tissues and improve the animals’ health span [185]. Moreover, we have found that G6PD transgenic mice have a larger muscle fiber size compared to the wild-type, while they age (manuscript under review). All these data highlight the importance that increasing the endogenous antioxidant defense has as a countermeasure to prevent disuse muscle atrophy.

Regarding the second approach, evidence exists both for and against the notion that by using antioxidants we can prevent disuse muscle atrophy. In 1991, a pioneering study that was subsequently confirmed [186,187], demonstrated for the first time that administration of vitamin E to rats protected against HU-induced muscle atrophy [108]. However, the vitamin E protection against disuse muscle atrophy, seemed to be achieved by the down-regulation of muscle proteolytic-related gene expression, rather than by its antioxidant properties [188]. The water-soluble analogue of vitamin E, Trolox, has also been widely used in research to counteract disuse muscle atrophy. It is generally considered that treatment of animals with Trolox protects the MV-induced diaphragmatic atrophy [77,160,189] and the loss of structural integrity in HDAC4mKO muscles following denervation [190] by decreasing oxidative stress. However, Trolox does not seem to protect against HU-induced muscle wasting [119].

Conflicting results have been published on the ability of NAC to protect against disuse muscle atrophy. NAC seems to be the most effective antioxidant in preventing respiratory muscle weakness and fatigue following exposure to chronic sustained hypoxia [191] and chronic intermittent hypoxia [192]. NAC also prevents MV-induced oxidative stress and protects the diaphragm against disuse-induced atrophy [112,113] (See section 3 for details). However, as in the case of Trolox, treatment with NAC does not protect against HU-induced muscle atrophy [193].

Mitochondrial targeted therapeutics are a promising tool in the prevention of inactivity-induced oxidative stress and in the protection of skeletal muscles against atrophy. Elamipretide (SS-31) is a synthetic peptide that concentrates in the inner mitochondrial membrane and selectively scavenges mitochondrial ROS [28]. Treatment of animals with SS-31 protects against MV-induced diaphragm disuse in rats [123,194] and HU-induced atrophy [195,196]. The mechanism of protection involves the prevention of protease activation (calpain and caspase-3) as well as a reduction in oxidative stress [28].

The Bowman-Birk inhibitor concentrate (soy protein with antioxidant properties) [197], resveratrol [198,199], and carotenoids (such as astaxanthin and β-carotene) [[200], [201], [202]] also exhibit protective effects against oxidative damage and muscle atrophy in muscle disuse models (including HU and denervation).

Allopurinol is a purine analogue and a very well-known inhibitor of XO widely used in the clinical practice for the management of gout and hyperuricemia [203]. In the early 90's it was found that the XO activity increase in immobilized muscle leading to oxidative stress and elevated antioxidant enzymes activity [145]. The efficacy of allopurinol in lessening the contractile dysfunction caused by HU in mice was reported by professor Reid's research group [146]. We observed that treatment with allopurinol prevents soleus muscle atrophy in hindlimb unloaded rats [55] but only partially protects against atrophy in HU mice and in lower leg immobilization following ankle sprain in humans [120]. The inhibition of XO activity with febuxostat [204] or allopurinol [181] in cancer cachexia also results in the conservation of skeletal muscle mass.

The synergistic effects of complex antioxidant cocktails on protection against disuse muscle atrophy have also been studied in rodents and in humans [205]. In a rodent study, an antioxidant cocktail (vitamin E, vitamin C and β-carotene) did not protect against hindlimb unloading-induced muscle atrophy [205]. We have recently tested the protective effect on the maintenance of muscle mass of a daily cocktail supplementation with Omega 3, selenium, polyphenols, and Vitamin E in healthy young subjects maintained for two months in HDBR at the MEDES space clinic in Toulouse [206]. We collected muscle biopsies before and after bedrest, and 10 days after remobilization. We did not find any protection in the loss of muscle mass and strength in the supplemented subjects.

These results underline the complexity of redox mechanisms and raise interrogations regarding the appropriate nutritional interventions to fight against muscle deconditioning. The redox modulation of muscle mass and function in disuse studies is dependent on muscle type, atrophy models, and even species (humans *vs* rodents). There is clear evidence in the literature showing that oxidative damage plays a causal role in diaphragm atrophy and dysfunction following MV. Accordingly, supplementation with antioxidants in this model have shown positive results. However, it is less clear whether oxidative stress is a cause of disuse atrophy in soleus or gastrocnemius muscle in HU mice and the literature reports conflicting results on the role of antioxidants on its prevention. The possibility that ROS production occurs at different extents and rates and that different mechanisms prevail in different models and muscles is consistent with the very variable rate of muscle atrophy [66]. Finally, limited evidence exists showing that administration of antioxidants have a positive impact in the prevention of the loss of muscle mass in ULLS, HDBR of DI in humans.

## 6 Conclusions

The mechanisms regulating muscle mass are relevant in several fields: healthy aging, diseases and sports medicine. The role of oxidative stress in disuse atrophy vary significantly through experimental conditions: atrophy models, species, and muscles. ROS exert opposite effects on muscle homeostasis in different conditions. Paradoxically, both skeletal muscle contraction and disuse or inactivity are associated with an increase in ROS generation leading to very different outcomes in the muscle cell. The amount of ROS, differences in the time course of ROS production, its compartmentalization, transience and even the nature of ROS can modulate their effects in muscle mass. The complexity of redox balance may explain why several studies have shown that some antioxidants can prevent inactivity-induced atrophy while others have been ineffective.

Targeting specifically the sources of skeletal muscle ROS generation with mitochondrial targeted scavengers or XOR inhibitors have shown promising results in the prevention of muscle atrophy. The experimental evidence provides molecular bases for interventions that by increasing the endogenous antioxidant defence (i.e G6PD) will delay the onset of disuse muscle atrophy, sarcopenia, and frailty.

## Author disclosure statement

The authors declare no competing financial interests.

## Declaration of competing interest

The Authors declare that there is no conflict of interest.
